# *Aerococcus mictus* as a cause of feline urinary tract infection: a case report

**DOI:** 10.1186/s12917-026-05462-3

**Published:** 2026-04-11

**Authors:** Annamaria Uva, Mattia Pirolo, Maria Alfonsa Cavalera, Giulia Tanas, Luca Guardabassi, Andrea Zatelli, Floriana Gernone

**Affiliations:** 1 Department of Veterinary Medicine, University of Bari, Valenzano, Italy; 2 Department of Veterinary and Animal Sciences, University of Copenhagen, Frederiksberg C, Denmark

**Keywords:** FLUTD, Infected urolithiasis, MALDI-TOF/MS, Whole-genome sequencing

## Abstract

**Background:**

Bacterial urinary tract infections (UTIs) are a noteworthy cause of lower urinary tract disease both in humans and companion animals, especially in patients with systemic comorbidities or underlying anatomical or functional urinary tract abnormalities. *Aerococcus* species are increasingly recognised as opportunistic uropathogens in human medicine; however, their role in UTIs in companion animals remains poorly characterised.

**Case presentation:**

The case of a neutered male European Shorthair cat with a history of antepubic urethrostomy and recurrent lower urinary tract signs, in which urine culture yielded a pure growth of *Aerococcus mictus*, is herein described. Species identification was achieved by whole-genome sequencing, which underscores the value of modern diagnostic tools in recognizing organisms that are often misclassified by conventional methods. The cat was successfully treated with targeted antimicrobial therapy.

**Conclusions:**

To the best of our knowledge, this is the first reported case of *A. mictus* as a causative agent of UTI in a cat, supporting its potential role as an opportunistic uropathogen in companion animals, highlighting the risk of misidentification among closely related *Aerococcus* species, and emphasizing the need for updated MALDI-TOF reference databases in veterinary microbiology.

**Supplementary Information:**

The online version contains supplementary material available at 10.1186/s12917-026-05462-3.

## Background

Urinary tract infection (UTI) refers to the adherence, multiplication and persistence of an infectious agent within the urogenital system that causes an associated inflammatory response and clinical signs [1]. The diagnosis of lower UTIs relies on the presence of lower urinary tract signs (LUTS) together with urinalysis findings and confirmation by quantitative bacterial urine culture [2]. Historically, bacterial UTIs were considered uncommon in cats, with early studies reporting a prevalence of < 3% among cats presenting with LUTS [2]; however more recent investigations indicate higher yet variable prevalence rates, ranging from 8% to 12% [3]. This variability likely reflects differences in study populations and diagnostic approaches, including case definitions, urine collection methods, and culture protocols. Prevalence is higher in older cats (over 10 years of age) likely due to the greater incidence of metabolic and systemic comorbidities [4]. Nonetheless, although bacterial UTIs are relatively uncommon in younger cats, the risk increases in the presence of predisposing local factors, including structural abnormalities, urolithiasis, chronic lower urinary tract disease, or iatrogenic factors [2]. Bacterial feline UTIs typically result from ascending entry of opportunistic bacteria from the host enteric or distal urogenital flora [2]. Among these, *Escherichia coli* is consistently reported as the most common isolated pathogen, accounting for 39–59% of positive urine cultures, followed by *Streptococcus* species (2–19%), *Enterococcus faecalis* (5–27%) and *Staphylococcus felis* (17–20%) [3, 5–9].

In recent years, emerging organisms associated with UTIs have gained increasing attention in human medicine. Notably, the genus *Aerococcus* (Gram-positive, α-haemolytic, catalase-negative cocci), specifically *A. urinae* and *A. sanguinicola*, has been recognized as opportunistic uropathogens, particularly in elderly patients with underlying urogenital diseases or structural/functional urological abnormalities [10]. With regard to *A. urinae*, recent taxonomic revisions have led to a proposed reorganization of the former *A. urinae* complex into distinct species. Among these, *A. mictus* has been differentiated from *A. urinae* on both genomic and phenotypic grounds [11]. Despite the growing clinical relevance of aerococcal UTIs in humans, reports of *Aerococcus* species as uropathogens in companion animals remain scarce and may be underestimated due to misidentification within the *Aerococcus urinae* complex. In this report, we describe the clinical presentation, diagnostic work-up, and management of an aerococcal UTI in a cat and provide definitive species-level identification of the isolate. Species assignment was confirmed by whole-genome sequencing, thereby documenting what appears to be the first published feline case of UTI caused by *Aerococcus mictus*.

## Case presentation

A four-year-old neutered male European Shorthair cat, weighing 4.4 kg, was presented for evaluation of stranguria, pollakiuria and haematuria of approximately three weeks duration.

At five months of age, the cat underwent an antepubic urethrostomy following a traumatic urethral rupture. Thereafter, the medical history was notable for recurrent culture-confirmed UTIs. The first episode occurred approximately six months post-urethrostomy and yielded *E. coli* on urine culture; a subsequent episode was associated with *E. faecalis*. In both instances, the isolates did not display antimicrobial resistance profiles, and clinical and microbiological resolution was achieved with antimicrobial therapy guided by susceptibility testing. Approximately 18 months before the current presentation, the cat underwent cystotomy for urolith removal; this episode was associated with a *Pasteurella multocida* UTI and likewise resolved following susceptibility-guided antimicrobial treatment. A further UTI episode occurred approximately six months before the current presentation, with *E. coli* isolated on urine culture and subsequent resolution with targeted antimicrobial therapy.

On admission, the patient was afebrile (rectal temperature 38.5 °C), with normal vital parameters, good nutritional status, and normal hydration status. Physical examination revealed severe urine scald dermatitis affecting the antepubic region surrounding the urethrostomy site, likely secondary to ongoing urinary leakage; no evidence of urethral obstruction was identified at presentation. The cat also appeared painful on abdominal palpation and showed marked discomfort on palpation of the skin lesion. Abdominal ultrasonography (A-FAST) showed a moderately filled urinary bladder with an irregular and mildly thickened wall. The bladder lumen contained suspended echogenic sediment, and two hyperechoic shadowing structures measuring approximately 8.3 and 5.8 mm, respectively, which were consistent with uroliths. Evaluation of the upper urinary tract did not reveal abnormalities. Two irregularly marginated radiopaque densities within the bladder lumen were confirmed by abdominal radiography, consistent with the presence of uroliths. Complete blood count (Siemens, ADVIA 2120, Erlangen, Germany) and biochemistry results (Beckman Coulter, Clinical Chemistry Analyzer AU680, Indianapolis, IN, USA) were within reference intervals; selected renal markers were urea 45 mg/dL (RI 35–76) and creatinine 1.2 mg/dL (RI 0.7–1.8). The urinalysis showed a specific gravity (USG) of 1.014, determined through refractometer measurement (Leica Vet 360, Misco Products Division, Cleveland, OH, USA). The low USG was interpreted as consistent with increased water intake, as the cat had been fed a wet diet with additional water supplementation since the onset of clinical signs. Dipstick analysis (Combur 9 Test, Roche, Rotkreuz, Switzerland) indicated a pH of 7.5 and marked haematuria. Urine sediment was prepared by centrifuging 5 mL of urine for 10 min at 900 × g and a 12 µL aliquot of the resuspended sediment was examined microscopically. Sediment examination confirmed marked haematuria (100 RBCs/hpf), along with pyuria (50 WBCs/hpf), struvite crystals, and numerous cocci (observed both extracellularly and within leukocytes on the cytospin-prepared, stained slide).

Urine obtained by ultrasound-guided cystocentesis was cultured using a semi-quantitative method with a calibrated 10 µl loop on 5% horse blood agar (Oxoid) and incubated at 37 °C for 48 h under aerobic conditions. No antibiotic treatment had been administered before the current urine culture; the last course of antimicrobial therapy dated back to the previous UTI episode, approximately six months earlier. After incubation, a pure and heavy growth (> 100,000 CFU per milliliter) of small α-haemolytic colonies was observed. Species identification by MALDI-TOF mass spectrometry (VITEK MS, bioMérieux) indicated *A. urinae* with 91.2% confidence (SARAMIS Knowledge base version 4.17). Antimicrobial susceptibility testing was performed by broth microdilution using Sensititre COMPGP1F plates (Thermo Fisher Scientific) in cation-adjusted Mueller-Hinton broth supplemented with 5% lysed horse blood (CAMHB-LHB; Oxoid), in accordance with the Clinical and Laboratory Standards Institute (CLSI) guidelines [12]. Because specific veterinary breakpoints for *Aerococcus* species are limited, results were interpreted using a combined approach. Interpretation was performed for penicillin, ampicillin, nitrofurantoin, tetracycline and vancomycin based on recently proposed CLSI M45 breakpoints [13], whereas results for all other antimicrobials were interpreted descriptively and are reported in Supplementary Table S1. No bacterial growth was observed in any antimicrobial-containing wells, whereas the growth control showed adequate growth, indicating inhibition at all tested antimicrobial concentrations. The strain was therefore considered fully susceptible to all antimicrobials included in the panel. The full MIC values are presented in Supplementary Table S1.

Whole-genome sequencing using the Illumina HiSeq platform, followed by de novo assembly of 586,903 paired-end reads with SPAdes with default parameters [14] and quality assessment with CheckM [15], yielded a 2.03-Mb genome (42.2% GC content; 99.97% completeness; 82× mean genome coverage) containing 1,835 coding sequences within 95 contigs (largest contig of 415.3 kbp; N50 of 215.8 kbp). Raw sequencing data have been deposited in the NCBI Sequence Read Archive under BioProject PRJNA1373523. Taxonomic assignment with PubMLST species identification [16] classified the isolate as *A. mictus*, with 98% support. Average nucleotide identity (ANI) analysis calculated with FastANI further supported this assignment, with the feline isolate being closer to the reference *A. mictus* genome Au-59-B15 (98.6% ANI), than the other *A. urinae* complex species, namely *A. urinae* reference strain CCUG 36,881 (92.5% ANI) [17], *A. loyolae* reference strain CDC-3352-U95 (93.9% ANI) and *A. tenax* reference strain UMB7049 (95.4% ANI). Notably, the ANI value relative to *A. mictus* exceeded the generally accepted 95–96% threshold for species delineation [18]. Screening for antimicrobial-resistance genes using ABRicate against both the ResFinder and CARD databases [19, 20] revealed no acquired resistance determinants, consistent with the phenotypic susceptibility profile. Similarly, no virulence determinants were identified by screening for virulence genes against the virulence factor database (VFDB) [21].

A diagnosis of infected urolithiasis, most consistent with struvite uroliths, was established based on urinalysis findings (alkaline urine pH, abundant struvite crystals), imaging results and urine culture; however, urolith composition was not analytically confirmed. The medical management plan consisted of susceptibility-guided antimicrobial therapy with oral amoxicillin/clavulanate (20 mg/kg every 12 h) for four weeks, combined with a wet diet and increased water intake to facilitate urolith dissolution. The prolonged antimicrobial course was chosen because the infection was complicated by concurrent urolithiasis, as bacteria may persist within or be released from infected uroliths during treatment [22].

At the 4-week follow-up, ultrasonography demonstrated a progressive reduction in the size of the uroliths which measured approximately 5.5 mm and 5.2 mm. Urinalysis revealed a specific gravity of 1.033 and a pH of 7.5, with persistent struvite crystalluria and haematuria but no evidence of pyuria, while urine culture was negative, thereby confirming resolution of the infection. In the light of the suboptimal USG and pH for struvite dissolution, review of the feeding history revealed poor dietary compliance, as the cat was receiving a mixed wet and dry diet not specifically formulated to acidify urine. Therefore, the ongoing therapeutic plan recommended stricter dietary management aimed at urine acidification, together with strategies to increase water intake, to be maintained until complete dissolution of the uroliths. Further follow-up beyond the reported re-evaluation was not available due to poor owner compliance, limiting confirmation of long-term outcome and complete urolith dissolution.

## Discussion and conclusions

This report describes a feline UTI caused by *A. mictus*, confirmed by whole-genome sequencing, and underscores that closely related *Aerococcus* species may be under-recognised in veterinary urinary diagnostics due to limitations of routine identification methods.

While regarded as opportunistic or low-virulence organisms, *Aerococcus* species have emerged in human medicine as clinically relevant causes of UTIs, particularly in elderly patients and those with chronic comorbidities or structural or functional urological abnormalities [10], such as those with urinary diversion procedures, prostatic disease, or indwelling catheters [23]. In the present case, *A. mictus* was isolated in pure culture from a cystocentesis sample, supporting its role as the primary etiologic agent. Nonetheless, the affected cat had substantial predisposing factors, including antepubic urethrostomy and concurrent urolithiasis [24], that likely impaired local host defence mechanisms, facilitating opportunistic colonisation by *A. mictus* and subsequent infection.

The increasing detection of *Aerococcus* species is attributed not only to an ageing population with increasing urological comorbidities but also to improved diagnostic accuracy. In this regard, *Aerococcus* species are easily overlooked due to their phenotypic similarity to other Gram-positive cocci and their slow growth in routine cultures, leading to historical underreporting [25]. Recent advances in clinical microbiology, including the widespread use of MALDI-TOF, the introduction of selective enrichment media, and the increasing use of molecular techniques, have improved the ability of diagnostic laboratories to detect uncommon or previously misclassified pathogens [26, 27]. In parallel, whole-genome sequencing has recently refined the taxonomy of pathogenic genera, including the genus *Aerococcus*, with a substantial diversity within what was historically grouped as *A. urinae* cluster [11]. These genomics insights have led to the reclassification of several isolates from human urinary samples that were initially identified as *A. urinae*, including the reassignment to newly delineated species such as *A. mictus* [28].

Although *A. urinae* has been associated in humans with a wide spectrum of clinical manifestations, ranging from asymptomatic bacteriuria to pyelonephritis, sepsis, and infective endocarditis [23], the clinical significance of *A. mictus* remains largely unknown. Its absence from clinical descriptions likely reflects its recent recognition and prior misclassification within the *A. urinae* complex. In the present report, the organism was initially identified as *A. urinae* by MALDI-TOF using SARAMIS Knowledge base version 4.17, where *A. mictus* is absent, whereas whole-genome sequencing confirmed it as *A. mictus*, highlighting the need to update MALDI-TOF reference spectrogram libraries to enable reliable discrimination of closely related *Aerococcus* species.

Evidence from clinical studies indicates that outcomes are most favourable when antimicrobial therapy is initiated promptly, whereas delayed identification or inadequate treatment increases the risk of systemic complications [29]. In the present case, the isolate exhibited full susceptibility to the antimicrobials tested, and targeted therapy resulted in rapid clinical improvement and microbiological clearance, as evidenced by a negative follow-up urine culture. The persistence of struvite crystalluria and uroliths despite infection resolution underscores the importance of addressing concurrent urinary tract abnormalities, as these factors may predispose to recurrent infections irrespective of the causative organism. This favourable response reinforces the principle that early organism-level identification and timely, appropriate antimicrobial selection are essential for managing opportunistic pathogens such as *A. mictus*, particularly in patients with underlying structural urinary tract alterations. Accordingly, *Aerococcus* species should be considered when Gram-positive cocci are observed in cystocentesis-derived urine from cats with LUTS or predisposing urinary tract abnormalities, and isolates should be identified to species level whenever feasible. In addition, molecular confirmation may be warranted when routine methods identify uncommon or unexpected uropathogens. However, veterinary data on *Aerococcus* species, and particularly on *A. mictus*, remain very limited. To the best of our knowledge, no previous reports are currently available in cats or, more broadly, in companion animals. Therefore, although findings from human medicine may help contextualize the possible clinical relevance of this isolate, their interpretation in a veterinary setting should remain cautious until further animal data become available.

We acknowledge some limitations. First, as a single case report, the findings cannot be generalised to the broader feline population, and the clinical relevance of *A. mictus* as a uropathogen in cats remains to be established through larger studies. Second, although the organism was isolated in pure culture from a cystocentesis sample, the presence of multiple predisposing factors, including antepubic urethrostomy and urolithiasis, complicates the assessment of its pathogenic role relative to opportunistic colonisation. Third, antimicrobial susceptibility results were interpreted only for few antimicrobials due to the lack of established veterinary-specific breakpoints for *Aerococcus* species. Finally, long-term follow-up was not available due to limited owner compliance, precluding assessment of recurrence risk and complete resolution of uroliths.

In conclusion, although based on a single clinical observation, this case suggests that *A. mictus* may be clinically relevant in feline urinary tract disease and raises the possibility that recently defined *Aerococcus* species could act as opportunistic pathogens in cats with underlying urogenital abnormalities. It also highlights the risk of misidentification of closely related *Aerococcus* species using routine diagnostic methods and underscores the need for increased clinical awareness and updated MALDI-TOF reference spectrogram libraries to accurately assess the clinical relevance of these organisms in veterinary medicine.

## Supplementary Information

Supplementary Material 1.

## Data Availability

Raw sequencing reads are available in the NCBI Sequence Read Archive under BioProject PRJNA1373523. Additional data supporting the findings of this study are available from the corresponding author upon reasonable request.
