# Supplementary material for: Aerococcus mictus as a cause of feline urinary tract infection: a case report
Source: BMC Vet Res. 2026 Apr 11;22:300. doi: 10.1186/s12917-026-05462-3 (PMC13196155; doi:10.1186/s12917-026-05462-3)
Supplement: Supplementary file 1 — Supplementary Material 1. [file 12917_2026_5462_MOESM1_ESM.docx]

**Table S1.** Minimum inhibitory concentration (MIC) values of the feline *Aerococcus mictus* strain.

| **Class** | **Antimicrobial** | **MIC (mg/L)** | **Intepretation^a^** |
| --- | --- | --- | --- |
| Aminoglycoside | Amikacin | ≤16 | - |
|  | Gentamicin | ≤4 | - |
| β-lactam | Amoxicillin/Clavulanic Acid | ≤0.25 | - |
|  | Ampicillin | ≤0.25 | Susceptible |
|  | Cefazolin | ≤2 | - |
|  | Cefovecin | ≤0.06 | - |
|  | Cefpodoxime | ≤2 | - |
|  | Cephalothin | ≤2 | - |
|  | Imipenem | ≤1 | - |
|  | Oxacillin | ≤0.25 | - |
|  | Penicillin | ≤0.06 | Susceptible |
| Chloramphenicol | Chloramphenicol | ≤8 | - |
| Fluoroquinolone | Enrofloxacin | ≤0.25 | - |
|  | Marbofloxacin | ≤1 | - |
|  | Pradofloxacin | ≤0.25 | - |
| Folate pathway inhibitors | Trimethoprim/Sulfamethoxazole | ≤2 | - |
| Glycopeptide | Vancomycin | ≤1 | Susceptible |
| Lincosamide | Clindamycin | ≤0.5 | - |
| Macrolide | Erythromycin | ≤0.25 | - |
| Nitrofuran | Nitrofurantoin | ≤16 | Susceptible |
| Rifamycin | Rifampin | ≤1 | - |
| Tetracycline | Doxycycline | ≤0.12 | - |
|  | Minocycline | ≤0.5 | - |
|  | Tetracycline | ≤0.25 | Susceptible |

^a^MIC values were interpreted according to ref. 13.
